# Multi-species alignments of *C. elegans lin-11* intronic sequences and putative transcriptional regulators

**DOI:** 10.1016/j.dib.2017.03.027

**Published:** 2017-03-18

**Authors:** Siavash Amon, Bhagwati P. Gupta

**Affiliations:** Department of Biology, McMaster University, Hamilton ON L8S-4K1, Canada

## Abstract

This data article contains multi-species alignments of the regulatory region of *C. elegans* LIM-HOX gene *lin-11* and lists of transcription factors that are predicted to bind to *lin-11* enhancers and regulate expression in amphid neurons. For further details and experimental findings please refer to the article by Amon and Gupta in Developmental Biology (S. Amon, B.P. Gupta, 2017) [1].

**Specifications Table**

**Table:** 

Subject area	*Biology*
More specific subject area	*Evolutionary Developmental Genetics*
Type of data	*Figure and Table*
How data was acquired	*Using software and web tools*
Data format	*Analyzed*
Experimental factors	*None*
Experimental features	*Genomic sequences were aligned and transcription factor binding sites were predicted*
Data source location	*Hamilton, Canada*
Data accessibility	*Data is with this article*

**Value of the data**

• *C. elegans lin-11* intron 3 possesses conserved sequence blocks that map within functionally defined neuronal enhancers.
• *C. briggsae lin-11* intron 3 possesses some sequences that are conserved in *C. nigoni* and *C. sinica* (two closest relatives of *C. briggsae*), but not in *C. elegans*.
• *In silico* analysis revealed putative transcription factor binding sites within conserved blocks of *C. elegans lin-11*. The functional relevance of these sites can be investigated to understand transcriptional regulation of *lin-11* in neuronal cell differentiation.

## 1 Data

**Fig. 1:**
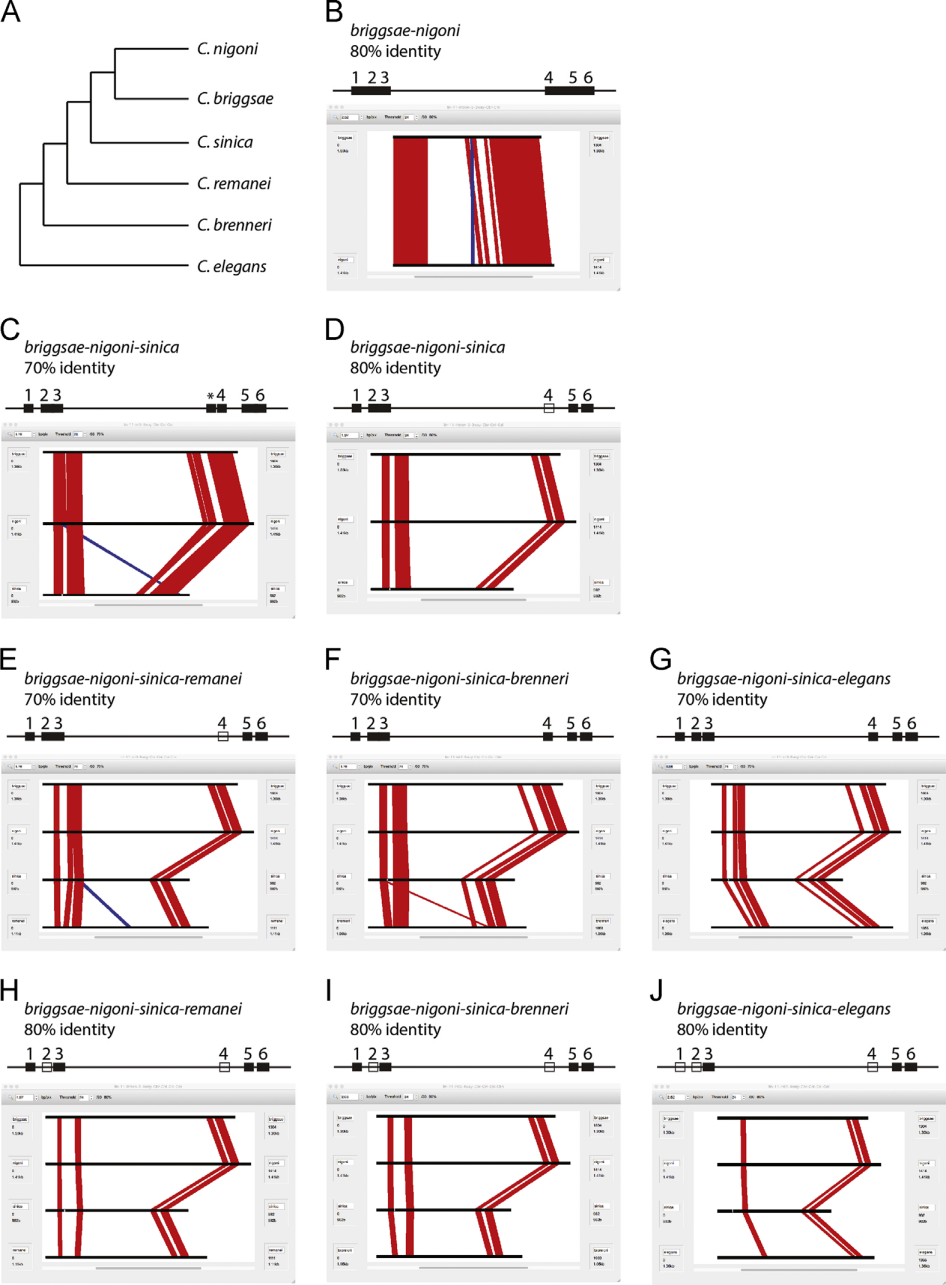
Multi-species sequence alignments of *lin-11* intron 3 using MussaGL. (A) Phylogenetic relationship of a subset of *Caenorhabditis* species. *C. nigoni* is the closest known relative of *C. briggsae,* followed by *C. sinica*. (B–J) Screenshots of aligned sequences. Species names and alignment threshold are shown above the each screenshot. Alignments in the same orientation are shown in red whereas those in the opposite orientation are in blue. At least six distinct conserved blocks (solid black rectangles on the horizontal line, drawn above the each screenshot) are observed in intron 3, termed 1 to 6 (each roughly 30–50 nucleotides long), that appear to form two distinct clusters. The blocks are not always distinct and some are part of larger conserved regions in 2-way and 3-way alignments. An open rectangle indicates an absence of a conserved block. Star in C marks an extra block that is not present in any other alignment.

Six *Caenorhabditis* species were used to perform sequence alignments of *lin-11* intron 3. These are *C. briggsae, C. sinica, C. nigoni, C. remanei, C. brenneri,* and *C. elegans*. MussaGL program (http://woldlab.caltech.edu/cgi-bin/mussa) was used at 70% and 80% window thresholds. Multiple alignments were carried out that included *C. briggsae* and *C. nigoni* (Fig. 1). In general, conservation decreases as the number of species and alignment threshold are increased. Four-way alignments reveal six distinct conserved blocks at 70% threshold. Some of these blocks are part of larger stretches in 2-way and 3-way alignments. At 80% threshold block 2 lacks conservation when either one of the *C. remanei, C. brenneri* and *C. elegans* species are included. Additionally, block 1 is lost in the case of *C. elegans*. Of the three sequence blocks described in the accompanied article [1], namely, C3-1, C3-2 and C3-3 that are conserved between *C. elegans, C. brenneri, C. remanei* and *C. briggsae*, block 3 corresponds to C3-1, block 5 to C3-2, and block 6 to C3-3 (Fig. 1).

We used a computational tool CIS-BP (http://cisbp.ccbr.utoronto.ca/TFTools.php) [2] to search for transcription factors (TFs) that may bind to conserved blocks in introns 3 (C3-1) and 7 (C7-1 and C7-2) and potentially *lin-11* expression in neurons. A total of 35 TF genes were identified for C3-1, 37 for C7-1, and 46 for C7-2 (Table 1). In addition, we searched for modENCODE dataset (http://www.modencode.org) and found eight TFs that bind to intron 7 sequences Table 1).

## 2 Experimental design, materials and methods

The *lin-11* intronic sequences from *Caenorhabditis* species were aligned using MussaGL (multi-species sequence analysis, version 1.1.0 for Mac OS X), an N-way sequence alignment software that was developed by Wold lab (Caltech, USA). The conservation threshold was set at 70% (21 per 30-nucleotide sliding window) and 80% (27 per 30-nucleotide sliding window).

To identify the putative TF genes for *C. elegans* introns 3 and 7, we used the CIS-BP database software. The setting included motif model ‘PWMs-LogOdds’ and Threshold 8. According to the website, this motif model option scores each position in each sequence with all position weight matrices, using a standard log odds scoring method. For more details see the help page on the website (http://cisbp.ccbr.utoronto.ca/help.html).

## Conflicts of interest

None.

## Appendix A Supplementary material

Supplementary material Excel file containing lists of putative *lin-11*-regulating TF genes based on CIS-BP searches and modENCODE Chip-Seq data..
